# Isolation of *Aloe saponaria*-Derived Extracellular Vesicles and Investigation of Their Potential for Chronic Wound Healing

**DOI:** 10.3390/pharmaceutics14091905

**Published:** 2022-09-08

**Authors:** Manho Kim, Ju Hyun Park

**Affiliations:** Department of Biomedical Science, Kangwon National University, Chuncheon-si 24341, Gangwon-do, Korea

**Keywords:** chronic wound healing, extracellular vesicles, *Aloe saponaria*, skin regeneration, inflammation, angiogenesis

## Abstract

A chronic wound is caused by a failure to progress through the normal phases of wound repair in an orderly and timely manner. To induce skin regeneration while inhibiting chronic inflammation, numerous natural products, and in particular, plant-derived biomaterials, have been developed. *Aloe saponaria*, is known to contain flavonoid and phenolic acid compounds with anti-oxidative and anti-inflammatory properties. Here, we isolated extracellular vesicles (EVs) from *Aloe saponaria* by polyethylene glycol (PEG)-based precipitation and investigated their potential as a therapeutic for chronic wound healing. The *Aloe saponaria*-derived EVs (AS-EVs) showed no significant cytotoxicity on several cell types, despite a high level of intracellular uptake. When lipopolysaccharide (LPS)-stimulated RAW264.7 macrophages were treated with AS-EVs, significant reductions in the expression of pro-inflammatory genes, such as interleukin-6 and interleukin-1β, were observed. Proliferation and migration of human dermal fibroblasts, as determined by the water-soluble tetrazolium salt-8 and transwell migration assay, respectively, were shown to be promoted by treatment with AS-EVs. It was also demonstrated that AS-EVs enhanced tube formation in human umbilical vein endothelial cells, indicating a stimulatory activity on angiogenesis; one of the crucial steps for effective wound healing. Collectively, our results suggest the potential of AS-EVs as a natural therapeutic for chronic wound healing.

## 1. Introduction

The skin is composed of several mechanically complex layers and participates in diverse functions, including temperature regulation and protection from the external environment [1]. Injuries to the skin, which acts as a physical barrier, can result in toxic exposures and bacterial infections that have the potential to cause further damage to internal organs [2]. Therefore, cutaneous wound healing consisting of coagulation/inflammation, re-epithelialization, and remodeling is an essential process for survival [3]. The first stage of cutaneous wound healing includes platelet-mediated blood clotting to prevent further invasion of foreign substances, and macrophage-mediated inflammation to remove foreign substances that have already entered the body [4]. However, if an abnormal immune response continues without prompt resolution at this stage, epidermal and dermal regeneration are inhibited and wound healing cannot be completed, resulting in a chronic wound [5,6]. Cases where anatomical and functional recovery of the skin is not completed within twelve weeks are termed chronic skin wounds, and these wounds, which require long-term treatment, are not only painful but create an economic burden to both the medical system and to patients [7,8]. Many researchers have sought to develop methods to effectively suppress the ongoing inflammation of chronic skin wounds in order to promote cutaneous wound healing [9,10,11]. In particular, the effects of natural products, such as honey and ginger, on cutaneous wound healing have been reported [12,13]. Meanwhile, several studies have shown that extracellular vesicles (EVs) obtained from various cell types not only have anti-inflammatory effects, but also promote cell proliferation and angiogenesis, which are necessary in the later stages of the wound-healing process [14]. These findings indicate that EVs represent a useful biomaterial for chronic wound healing.

EVs play a crucial role in cell-to-cell communication by delivering biomolecules, such as proteins, nucleic acids, and even pharmacological compounds [15]. Among EVs of various sizes (100–1000 nm), exosomes smaller than 150 nm can penetrate deep into tissues and serve as intercellular communication mediators [16]. Exosomes isolated from various mammalian cells exhibit many biological functions, including anti-inflammatory, anti-aging, and anti-oxidative effects [17,18,19,20]. In several recent studies, EVs were also used as carriers for protein and nucleic acid, as well as drug delivery, due to their targeting properties, low toxicity, and prolonged circulation time [21]. However, for practical clinical applications requiring massive quantities, mammalian cell-derived EVs (MDEVs) are limited by their high cost and low productivity. In addition, the requirement for xeno-free culture conditions and strict purification standards due to undefined and animal-derived substances, that may be present in mammalian cell culture, is another limitation of MDEVs for use in clinical applications [22]. Accordingly, interest in plant-derived EVs (PDEVs) as a replacement for MDEVs is rapidly growing. It has been reported that PDEVs play a role in unrelated interspecies cell-to-cell communication and have a structural composition similar to MDEVs [23,24,25]. Encouragingly, anti-oxidative and therapeutic effects of PDEVs isolated from various plants, such as cabbage, grape, carrot, and lemon, have recently been reported [22,26,27,28].

Despite remarkable advances in the development of EV isolation methods over the past decade, there is still a need to establish simpler and more efficient isolation protocols. Until recently, ultracentrifugation-based methods were widely used to isolate EVs [29] despite having a number of drawbacks, which include a tendency for vesicle aggregation, impairment of integrity due to high centrifugation speeds, as well as the need for expensive equipment [29,30]. To overcome these shortcomings, other EV isolation methods involving immuno-affinity and size exclusion chromatography have been developed [31]. However, these are limited in terms of scale because the sample volumes that can be handled are restricted [31,32]. EVs’ isolation via tangential flow filtration (TFF) is an emerging strategy for process scale-up [33,34]. TFF allows the fluid containing EVs to flow tangentially across the membrane, thereby avoiding the clogging issue due to the accumulation of large particles on the filter membrane [35,36]. However, this method also has some limitations, including the need for special equipment, such as a pump system and filter modules. On the other hand, polymer-based precipitation is regarded as a relatively simple method for isolating EVs without special equipment [37]. Polyethylene glycol (PEG)-based precipitation can isolate EVs at physiological pH ranges, while preserving their integrity and allowing the processing of large sample volumes at low cost [29]. Since sample volumes can range from tens of milliliters to liters in a typical batch isolation of PDEVs, PEG-based precipitation is considered a promising method for large-scale isolation of PDEVs if the procedure can be optimized, despite it having generally lower purity than other protocols. PEG, which is widely used for conjugation with various drugs such as proteins and lipid nanoparticles, is one of the biocompatible polymers approved by the U.S. Food and Drug Administration (FDA) as it has been evaluated as less toxic and safe to the human body [38,39]. This also indicates the suitability of PEG-based precipitation as an EV isolation method for further clinical applications of PDEVs.

The genus *Aloe* consists of more than 550 species, and the leaves and roots of *Aloe* contain various phytochemicals [40]. The *Aloe* family, including *Aloe vera* and *Aloe arborescens*, is well known for their medicinal properties with antioxidant, anti-inflammatory, anti-bacterial, anti-aging, and anti-cancer effects having been demonstrated [41,42,43,44,45,46]. Among the numerous *Aloe* species, *Aloe saponaria* (*syn. Aloe maculata*) has been used to treat burns in southern Brazil [47]. Like *Aloe vera*, one of the most studied *Aloe* species, *Aloe saponaria* contains flavonoids (rutin, quercetin, and kaempferol) and phenolic acids (gallic acid and caffeic acid) with anti-inflammatory and anti-oxidative activities [48,49,50]. *Aloe saponaria* mannan can inhibit the activation and proliferation of cancer cells, and the anti-nociceptive and anti-inflammatory effects of *Aloe saponaria* extract in models of thermal burns in rats have been reported [48,51]. Herein, we report the isolation of *Aloe saponaria*-derived EVs (AS-EVs) and the results obtained by investigating their therapeutic potential for chronic wound healing using in vitro experimental models. The results of our study suggest that AS-EVs show promise as a natural biomaterial for chronic wound healing.

## 2. Materials and Methods

### 2.1. Isolation of AS-EVs from Aloe saponaria Peels

Polyethylene glycol (PEG) 6000 (Sigma-Aldrich, St. Louis, MO, USA) was dissolved in 1 M sodium chloride (LPS Solution, Daejeon, Korea) solution to prepare a two-fold concentrated stock solution. *Aloe saponaria* was purchased from a local market in the Republic of Korea. Whole *Aloe saponaria* was washed three times with distilled water to remove impurities such as dirt and dust. After separating from the inner gel part by knife, the *Aloe saponaria* peel was mixed with chilled phosphate-buffered saline (PBS) at a ratio of 1:3 (*w*/*w*). The mixture was homogenized with a kitchen blender, and the *Aloe saponaria* pulp was sequentially centrifuged at 1000× *g* for 10 min, 3000× *g* for 30 min, and 10,000× *g* for 60 min to remove large debris. Following filtration through a 0.45 μm filter (Hyundai Micro, Seoul, Korea), the supernatant was mixed with an equal volume of 2× PEG stock solution and incubated at 4 °C for the desired time. Precipitated samples were collected by centrifugation at 1500× *g* for 30 min and the supernatant was discarded. To remove the excess PEG, the conical tubes (SPL, Pocheon, Korea) were turned upside down for 3 to 5 min and washed gently with PBS, being careful not to disturb the pellet. The pellet containing AS-EVs was suspended in PBS and stored at −80 °C until used.

### 2.2. Characterization of AS-Evs

The concentration and size distribution of AS-EVs were determined using nanoparticle tracking analysis (NTA, NanoSight NS300, Malvern Panalytical, Malvern, UK). The total protein concentration in the isolated AS-EVs samples was quantitively analyzed by a BCA assay (Thermo Fisher Scientific, Waltham, MA, USA). Next, the morphologies of AS-EVs were observed by transmission electron microscopy (TEM). AS-EVs were fixed with 4% paraformaldehyde (PFA, Sigma-Aldrich, St. Louis, MO, USA) at 4 °C for 16 h, and then dried on a carbon-coated copper mesh grid (Ted Pella, Inc., Redding, CA, USA). After washing three times with PBS, AS-EVs on copper mesh grids were stained with 1% phosphotungstic acid (Sigma-Aldrich, St. Louis, MO, USA). To remove the staining reagent, the copper mesh grid was washed three times with distilled water and then dried for another 15 min. The dried sample was observed through a JEM-2100F electron microscope (JEOL, Tokyo, Japan).

### 2.3. Cell Culture

RAW 264.7 cells were obtained from the Korea Cell Lines Bank (Seoul, Korea) and maintained in Dulbecco’s modified Eagle’s medium (DMEM, Welgene, Daejeon, Korea), supplemented with 10% fetal bovine serum (FBS, Welgene), 100 U/mL penicillin, and 100 μg/mL streptomycin (1% PS, Thermo Fisher Scientific, Waltham, MA, USA). RAW 264.7 cells were detached using a cell scraper (SPL) and passaged at a 1:6 splitting ratio. Human dermal fibroblasts (HDFs, #C-004-5C, Thermo Fisher Scientific, Waltham, MA, USA) were cultured in DMEM supplemented with 10% FBS and 1% PS. When confluency reached 80%, cells were detached by Trypsin-EDTA (TE, Thermo Fisher Scientific) treatment and passaged at a 1:8 ratio. Human umbilical vein endothelial cells (HUVECs, #C-003-5C, Thermo Fisher Scientific) isolated from the umbilical vein were cultured in Endothelial Cell Growth Medium-2 (EGM-2, Lonza, Basel, Switzerland). At 80% confluence, the cells were dissociated with TE and plated onto gelatin-coated plates at a 1:4 sub-cultivation ratio. All cells were maintained at 37 °C in a humidified atmosphere containing 5% CO_2_.

### 2.4. Cytotoxicity Assessment of AS-EVs

To evaluate the cytotoxicity of AS-EVs, cells were aliquoted into 24-well plates (SPL) at a density of 1 × 10^4^ cells/cm^2^. After incubation for 24 h, cells were washed with PBS and incubated for 24 h and 48 h in culture medium supplemented with AS-EVs at different concentrations, ranging up to 5 × 10^9^ particles/mL. Thereafter, the cells were stained with trypan blue solution (Thermo Fisher Scientific, Waltham, MA, USA) and the cell viability was measured by counting the number of viable and dead cells with a hemocytometer.

### 2.5. Intracellular Delivery of AS-EVs

To observe intracellular delivery of AS-EVs, vesicles were labeled with PKH-67 green fluorescent dye (Sigma-Aldrich, St. Louis, MO, USA) according to the manufacturer’s instructions. HDFs were treated with 5 × 10^9^ particles/mL of the labeled AS-EVs for 8 h, and the nuclei were subsequently stained with 2.5 μg/mL of Hoechst 33342 (Thermo Fisher Scientific, Waltham, MA, USA) for 20 min. Intracellularly delivered PKH-67-labeled AS-EVs were then visualized under a fluorescence microscopy (Leica, Bensheim, Germany).

### 2.6. Reverse Transcriptase Polymerase Chain Reaction

To evaluate the effects of AS-EVs on inflammatory response, RAW 264.7 cells were plated in 24-well plates at a density of 1.5 × 10^5^ cells/cm^2^ and incubated for 24 h. Cells were then further incubated in a medium supplemented with different concentrations of AS-EVs for 24 h. After stimulation of inflammatory response by treatment with 50 ng/mL lipopolysaccharide (LPS, Sigma-Aldrich, St. Louis, MO, USA) for 24 h, total RNA was isolated from the harvested cells using RiboEx™ LS (GeneAll, Seoul, Korea), according to the manufacturer’s instructions. Next, the purified total RNA was reverse transcribed using TOPScript™ RT DryMIX (Enzynomics, Daejeon, Korea) with a dT 18 plus primer. For reverse transcriptase polymerase chain reaction (RT-PCR) analysis, the synthesized cDNA was mixed with specific primers and 2× TOPsimple™ DyeMIX-HOT (Enzynomics) and amplified for 25 cycles using a T100 Thermal Cycler (Bio-Rad, Hercules, CA, USA). Quantitative real-time polymerase chain reaction (qPCR) analysis was performed using a qTOWER³ machine (Analytik Jena, Jena, Germany) by mixing the synthesized cDNA with specific primers and TOPreal™ qPCR 2× PreMIX (SYBR Green with low ROX, Enzynomics). The sequences of specific primers used are summarized in Table 1. The expression level of each pro-inflammatory mRNA was normalized to that of glyceraldehyde 3-phosphate dehydrogenase (GAPDH) as an endogenous control, and the relative mRNA expression level compared to non-stimulated cells was determined by the 2^−ΔΔCt^ method.

### 2.7. Cell Proliferation and Migration Assay

The effect of AS-EVs on the proliferation and migration of HDFs was evaluated by the water-soluble tetrazolium salt-8 (WST-8) and transwell migration assay, respectively. In the WST-8 assay, HDFs were plated in 96-well culture plates (SPL) at a density of 0.5 × 10^4^ cells/cm^2^ and incubated for 24 h. Following 24 h incubation in DMEM containing 0.5% FBS for serum starvation, cells were treated with different concentrations of AS-EVs in serum-free DMEM/F12 for 48 h. After addition of WST-8 solution (Biomax Inc., Seoul, Korea) and further incubation for 2 h, an absorbance at 450 nm was measured using a microplate reader. Transwell migration assays were performed as described previously [19,20]. Briefly, HDFs were plated on filters in the upper chamber of a transwell plate (Corning, Glendale, AZ, USA), with 8.0 μm pores at a density of 4 × 10^4^ cells/cm^2^ and incubated for 24 h. Then, culture media were replaced with serum-free DMEM/F12 supplemented with AS-EVs and incubated for another 24 h to induce migration to the bottom side of the membrane. After fixation with 4% PFA, cells remaining on the upper side were removed using a cotton swab. Thereafter, cells on the bottom side of the membrane were stained with 0.5% crystal violet (CV, Sigma-Aldrich) for 15 min and observed under an optical microscope. For quantification of CV staining, the stained membrane was immersed in 50% acetic acid (DAEJUNG, Siheung, Korea) to dissolve the crystalized CV, and then the optical absorbance at 560 nm was measured using a microplate reader.

### 2.8. Tube Formation Assay

To evaluate the effect of AS-EVs on capillary-like tube formation, 150 μL of Matrigel (Corning) was added to each 48-well plate (SPL) and gelled at 37 °C for 30 min. Then, HUVECs were plated in the pre-coated wells at a density of 2.5 × 10^4^ cells/cm^2^ and incubated in EGM-2 medium, containing different concentrations of AS-EVs. After 16 h, the four fields of each well plate were randomly imaged with an inverted microscope (Leica), and the total tube lengths of each image were quantified using ImageJ software (National Institute of Health, Bethesda, MD, USA).

### 2.9. Statistical Analysis

The results of this study are expressed as mean ± standard deviation, and statistical significance between the experimental groups was determined by one-way analysis of variance (ANOVA) with Tukey’s post hoc test. A value of *p* < 0.05 was considered statistically significant. All quantitative data shown in this study were obtained from at least triplicate samples in a representative of experiments conducted several times.

## 3. Results and Discussion

### 3.1. Isolation of AS-EVs by PEG-Based Precipitation

**Figure 1:**
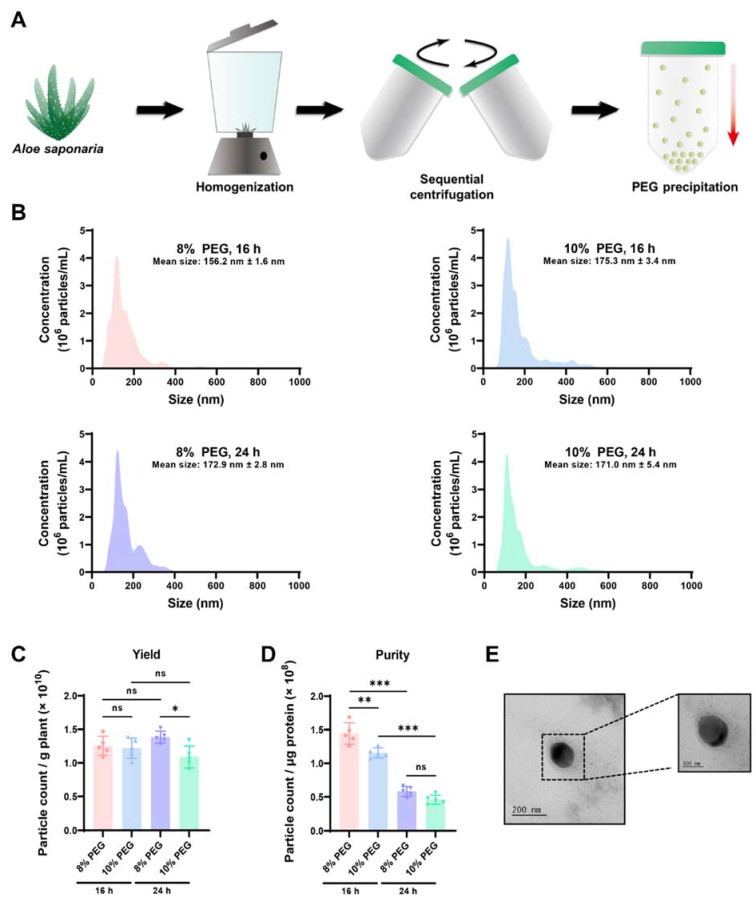
Isolation of PDEVs from *Aloe saponaria* peels. (**A**) Schematic illustration of EV isolation procedure from *Aloe saponaria*. (**B**) Size distribution of AS-EVs precipitated for the indicated time using 8% and 16% PEG solutions, as determined by NTA. In all cases, AS-EVs showed a heterogenous size distribution, and the mean size was found to be less than 200 nm. (**C**) Yield of AS-EVs for each precipitation condition. The yield was determined by dividing the total count of isolated vesicles by the mass of *Aloe saponaria* extract (* *p* < 0.05, ns: not significant, *n* = 5). (**D**) Purity of AS-EVs for each precipitation condition. The purity is represented as the count of particles-to-microgram of total protein (** *p* < 0.01, *** *p* < 0.005, ns: not significant, *n* = 5). (**E**) Transmission electron microscopy image of isolated AS-EVs.

Ultracentrifugation and size exclusion chromatography are representative techniques for the isolation of high-purity EVs from a variety of sources [22,52,53]. However, considering the large sample volumes that need to be processed in order to scale up, the PEG-based precipitation method, which can easily and reproducibly handle large sample sizes at a low cost, is considered to be a promising strategy for the isolation of PDEVs [29]. Therefore, we optimized a PEG-based precipitation method to efficiently isolate EVs from *Aloe saponaria* peels (Figure 1A). Following sequential centrifugation for the removal of large impurities, *Aloe saponaria* juice was mixed with various concentrations of PEG and incubated at 4 °C for 16 and 24 h, respectively. Thereafter, to determine the optimal conditions for AS-EVs isolation, the size distribution of isolated AS-EVs was analyzed using NTA. In all cases, AS-EVs showed multiple peaks, indicating size heterogeneity, with an overall average size of less than 200 nm (Figure 1B). Based on counting, AS-EVs isolated by each protocol showed no significant differences in yield from the same starting mass of *Aloe saponaria* extract (Figure 1C). Next, we evaluated the purities of each sample through the particle count-to-protein ratio. Trace impurities, including PEG, remaining even after the wash step may have affected the quantification of total proteins, leading to distortions in AS-EV purity calculations. Nevertheless, it is considered that the purity of AS-EVs isolated by the different protocols used in this study can be compared with each other. AS-EVs isolated with 8% PEG and incubated for 16 h showed the highest purity, although the effect of PEG concentration was relatively insignificant compared to the incubation time (Figure 1D). TEM images demonstrated a spherical morphology for AS-EVs (Figure 1E). Accordingly, in subsequent experiments, we used AS-EVs isolated with 8% PEG and incubated for 16 h.

### 3.2. Cytotoxicity and Intracellular Delivery of AS-EVs

**Figure 2:**
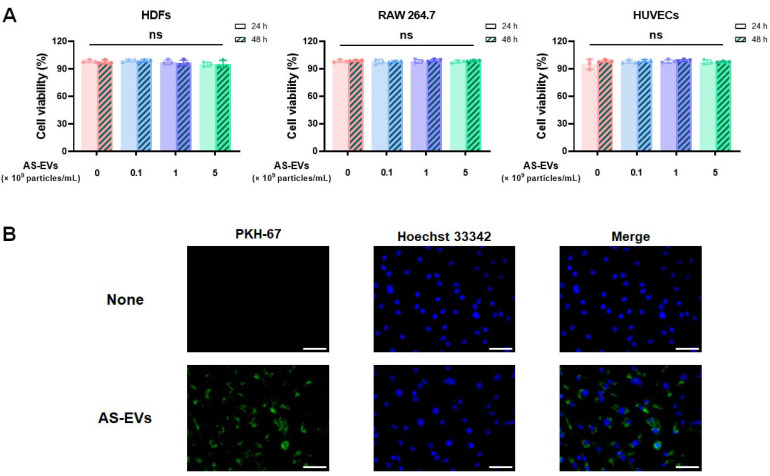
Cytotoxicity and intracellular delivery of AS-EVs. (**A**) Cytotoxicity of AS-EVs in HDFs, RAW 264.7 cells, and HUVECs, respectively. After 24 h and 48 h treatment with AS-EVs, viability was measured by trypan blue exclusion assay. (ns: not significant, *n* = 3) (**B**) Uptake of AS-EVs into HDFs. HDFs were incubated for 8 h in a growth medium containing PKH-67 green fluorescent dye-labeled AS-EVs, and then observed under a fluorescence microscope. Scale bar indicates 100 μm.

To evaluate cytotoxicity, we added AS-EVs to three types of cells involved in the wound-healing process. HDFs located in the dermis are a major cell type contributing to reconstitution of damaged dermal tissue [22,54], HUVECs are an endothelial cell that plays a crucial role in angiogenesis [55,56], and RAW264.7 is an immortalized mouse macrophage cell line [22,57,58]. No significant cytotoxicity was observed across the entire dose and incubation time range in all three cell types, even after treatment with AS-EVs up to 5 × 10^9^ particles/mL for 24 and 48 h, respectively (Figure 2A). As shown in Figure 1D, AS-EVs isolated via PEG-based precipitation had a relatively lower purity compared to the MDEVs isolated with ultracentrifugation and size exclusion chromatography [20,59,60]. In terms of the clinical application of MDEVs isolated from in vitro cell culture, a major concern is the need to completely remove undefined compounds derived from host cells or animal serum that may be immunogenic and/or pathogenic [61,62]. PDEVs, in contrast, are composed of intracellular molecules from plants that are consumed for nutrition and considered to have low cytotoxicity for both in vitro and in vivo applications [22,63,64,65,66]. It has been demonstrated that the oral administration of ginger-derived PDEVs to mice resulted in no significant toxicity or side effects, and grapefruit-derived PDEVs that do not cross the placenta can be used as a drug delivery vehicle for pregnant women without any significant side effects [64,65,66]. Collectively, AS-EVs isolated via the PEG-based precipitation, which have been shown to be non-toxic to mammalian cell types used in this study, are not considered to have the potential for severe side effects or toxicity in various applications as the preparations derive from an edible plant. On the other hand, the potential risks posed by impurities, including PEG, even in trace amounts, should be considered prior to further clinical applications, although many studies have demonstrated the biocompatibility of PEGs. Improving the PEG-based precipitation protocol, for example by adding multiple wash steps using optimized buffers, would be helpful to minimize the risks from impurities.

EVs can deliver biomolecules, such as proteins and nucleic acids, into mammalian cells by fusing directly with the plasma membrane or via various endocytic routes, including clathrin-mediated and caveolin-dependent endocytosis. [67]. To confirm intracellular delivery, HDFs were treated with 5 × 10^9^ particles/mL of AS-EVs labeled with PKH-67 dye for 8 h, and subsequently observed under a fluorescence microscope (Figure 2B). Green fluorescence within HDFs indicated intracellular uptake, and thus demonstrated that AS-EVs could deliver functional biomolecules derived from *Aloe saponaria* into mammalian cells.

### 3.3. Effect of AS-EVs on Expression of Pro-Inflammatory Cytokines

**Figure 3:**
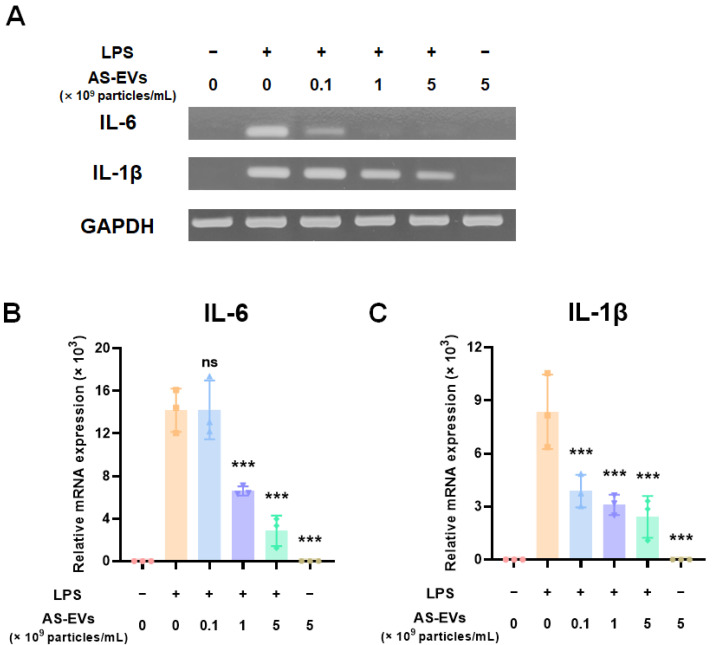
Effect of AS-EVs on expression of pro-inflammatory cytokines in LPS-stimulated RAW 264.7 cells. (**A**) RT-PCR analysis of IL-6 and IL-1β mRNA expression in AS-EV-treated cells followed by induction of inflammatory response by LPS treatment. (**B**,**C**) The mRNA expression levels IL-6 and IL-1β in AS-EV-treated cells were quantitatively analyzed by qPCR analysis. AS-EVs reduced the expression of LPS-induced pro-inflammatory mRNAs. Statistical significance was determined by comparison with the control group not treated with AS-EVs (*** *p* < 0.005, ns: not significant, *n* = 3).

Inflammation plays a crucial role in preparing for re-epithelialization during cutaneous wound healing [68,69]. In acute wound healing, the inflammatory response can promote the proliferation and migration of fibroblasts through the clearance of foreign substances, such as invading pathogens [5]. However, prolonged inflammatory responses can lead to chronic skin wounds by impairing the transition from inflammation to re-epithelialization [6,70]. Therefore, we first assessed the effect of AS-EVs on the expression of pro-inflammatory cytokines to evaluate their potential for chronic wound healing. After treatment with up to 5 × 10^9^ particles/mL of AS-EVs, an inflammatory response of RAW 264.7 was stimulated with LPS treatment [71]. Then, the mRNA expression levels of pro-inflammatory cytokines, such as interleukin (IL)-6 and IL-1β, were analyzed by RT-PCR analysis [72,73]. The results of the semi-quantitative RT-PCR analysis indicated that the AS-EVs exhibited no significant effect on inflammation, but rather inhibited the mRNA induction of pro-inflammatory cytokines, caused by LPS (Figure 3A). qPCR analysis also demonstrated that AS-EVs inhibited the inflammatory response induced by LPS treatment. The mRNA expression levels of IL-6 and IL-1β increased more than 14,000- and 8000-fold, respectively; however, treatment with 5 × 10^9^ particles/mL of AS-EVs showed more than 70% inhibition in both cases. (Figure 3B,C). These results suggest that suppression of excessive and continuous inflammation by AS-EVs could promote chronic wound healing by inducing a transition from inflammation to re-epithelialization.

### 3.4. Effect of AS-EVs on Proliferation and Migration of HDFs

**Figure 4:**
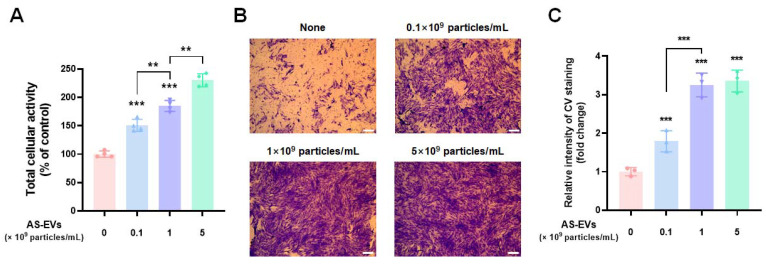
Effect of AS-EVs on the proliferation and migration of HDFs. (**A**) After serum starvation, HDFs were treated with AS-EVs for 48 h in serum-free DMEM/F12. The population of viable cells was evaluated by WST-8 assay. Statistical significance was determined by comparison with the control group not treated with AS-EVs if not indicated (** *p* < 0.01, *** *p* < 0.005, *n* = 4). (**B**) Migration of HDFs treated with AS-EVs was evaluated by transwell migration assay. After 24 h treatment with AS-EVs, the HDFs that had migrated to the bottom side of the porous membrane were stained with CV and followed by fixation with 4% PFA. Scale bar indicates 200 μm. (**C**) For quantitative analysis, the stained membrane was cut out and immersed in a 50% acetic acid solution to dissolve the crystalized CV. The intensity of CV staining was determined as optical absorbance at 560 nm. Statistical significance was determined by comparison with the control group not treated with AS-EVs (*** *p* < 0.005, *n* = 3).

When an inflammatory response subsides, proliferation and migration of skin cells such as fibroblasts and keratinocytes are key steps in successful cutaneous wound healing [70]. Unlike normal wounds, chronic skin wounds are characterized by increased activity of matrix metalloproteinases (MPPs) that degrade extracellular matrix (ECM) proteins and growth factors [74,75,76]. The overactivation of MPPs inhibits fibroblast proliferation and the subsequent recovery of wound sites by forming a proteolytic environment that is unfavorable for healing [77,78]. Therefore, promotion of fibroblast proliferation is an important task for the restoration of the impaired wound. To evaluate the effect of AS-EVs on the proliferation of fibroblasts, HDFs (a cell type widely used in cutaneous-wound-healing studies in vitro [79,80]), were treated with up to 5 × 10^9^ particles/mL of AS-EVs after serum starvation. WST-8 analysis after 48 h of AS-EV treatment demonstrated that the proliferation of HDFs was increased in a dose-dependent manner (Figure 4A). Notably, the group treated with 5 × 10^9^ particles/mL exhibited a 2.3-fold increase in proliferation compared to the non-treated control group.

We next investigated the effect of AS-EVs on the migration of HDFs by a transwell migration assay. HDFs plated on the upper membrane of the transwell can migrate to the opposite side of the membrane, and migration can be detected by staining cells on the bottom with CV [19]. The results revealed that migration of HDFs was significantly promoted by treatment with AS-EVs at various concentration up to 5 × 10^9^ particles/mL of AS-EVs (Figure 4B). Quantitative analysis of CV staining also demonstrated that the stimulatory effect was increased by AS-EVs, and the migration of cells treated with 1 × 10^9^ particles/mL was increased more than three-fold compared to the non-treated controls (Figure 4C). Taken together, our results indicate the potential of AS-EVs to accelerate the wound-healing process by promoting the proliferation and migration of HDFs.

### 3.5. Effect of AS-EVs on Angiogenesis

**Figure 5:**
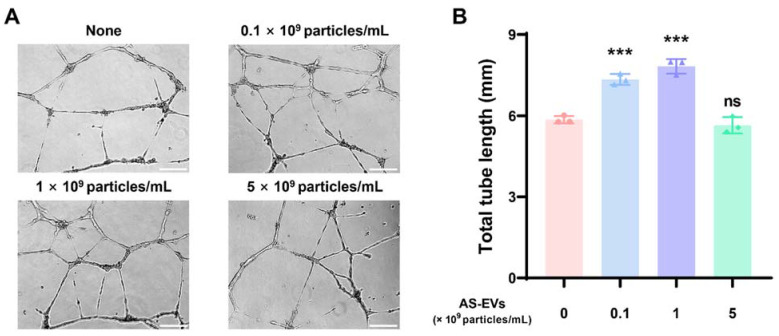
Effect of AS-EVs on the capillary-like tube formation of HUVECs. (**A**) Representative image of HUVEC tube formation. HUVECs plated on Matrigel-coated wells were treated with AS-EVs for 16 h in EGM-2 medium. Scale bar indicates 200 μm. (**B**) Total tube length (mm) was measured using ImageJ software. Statistical significance was determined by comparison with the control group not treated with AS-EVs (*** *p* < 0.005, *n* = 3).

Angiogenesis at the wound site is known to be a vital process for normal wound healing [81,82]. Newly formed blood vessels can promote fibroblast proliferation, ECM synthesis, and re-epithelialization by transporting oxygen and nutrients to the wound site [83,84]. Accordingly, several studies have attempted to repair chronic skin wounds by promoting angiogenesis [85,86,87]. The capillary-like tube formation assay using endothelial cells on a basement membrane matrix, such as Matrigel, is a useful in vitro experimental method for evaluating angiogenesis [88]. For tube formation analysis, HUVECs were plated on gelled Matrigel, and then treated with AS-EVs at various concentrations. From microscopic observations, significant increases in tube formation were observed at both 0.1 and 1 × 10^9^ particles/mL of AS-EVs. Interestingly, the effect was somewhat reduced at the higher dose of 5 × 10^9^ particles/mL (Figure 5A). The tube formation of endothelial cells correlates with physicochemical interactions between cells and basement membrane matrices [89,90], and some recent studies have reported that EVs play a role in cell–matrix interactions [91,92]. Therefore, it is possible that too many EVs contained in the medium can interfere with adequate cell–matrix interactions, thereby reducing the stimulatory effect on the tube formation of HUVECs. The total tube length of the non-treated control cells analyzed by ImageJ software was 5.85 ± 0.14 mm; however, that of the cells treated with 0.1 and 1 × 10^9^ particles/mL of AS-EVs were 7.33 ± 0.20 mm and 7.82 ± 0.27 mm, respectively (Figure 5B). These results clearly demonstrate the angiogenic effect of AS-EVs.

## 4. Conclusions

Several studies have reported that extracts of *Aloe* species not only show anti-inflammatory effects, but also promote angiogenesis and skin cell proliferation, which are required for chronic wound healing [47,48,93,94]. Considering that PDEVs contain biomolecules such as phytochemicals, nucleic acids, and proteins, it is suggested that PDEVs derived from *Aloe* species can also promote wound healing. Recently, the antioxidant effect of EVs derived from *Aloe vera*, a representative *Aloe* species, has been reported [54]. In the present study, we aimed to isolate PDEVs from *Aloe saponaria* through an optimized PEG-based precipitation protocol and investigate their potential for chronic wound healing. Given the use of edible plants with low toxicity, PEG-based precipitation can rapidly and inexpensively isolate large amounts of PDEVs, and it appears to be a promising strategy for PDEV isolation. Despite clear intracellular uptake, no cytotoxicity was observed when various types of mammalian cells were treated with AS-EVs. In addition, AS-EVs attenuated the expression of pro-inflammatory cytokines, which is the first step in chronic skin wound healing, and promoted the proliferation and migration of dermal fibroblasts, which are necessary subsequent steps. The tube formation assay using endothelial cells revealed the angiogenesis-promoting effect of AS-EVs, indicating that this preparation has the potential to accelerate the chronic-wound-healing process by facilitating efficient nutrient transfer to the wound site. Taken together, our results not only demonstrate the utility of isolating PDEVs by PEG-based precipitation, but also show the potential of AS-EVs as a natural biomaterial for chronic skin wound healing.

## Figures and Tables

**Table 1:** List of specific primers for PCR.

Gene	Primer	Sequence (5′-3′)
GAPDH	Sense	GTG GCA AAG TGG AGA TTG TTG
	Antisense	CTC CTG GAA GAT GGT GAT GG
IL-6	Sense	GCT ACC AAA CTG GAT ATA ATC AGG A
	Antisense	CCA GGT AGC TAT GGT ACT CCA GAA
IL-1β	Sense	AGT TGA CGG ACC CCA AAA G
	Antisense	AGC TGG ATG CTC TCA TCA GG

## Data Availability

The original contributions presented in the study are included in the article.
